# Epidemiological characteristics, baseline clinical features, and outcomes of critically ill patients treated in a coronavirus disease 2019 tertiary center in continental Croatia

**DOI:** 10.3325/cmj.2022.63.6

**Published:** 2022-02

**Authors:** Marcela Čučković, Željka Drmić, Marko Pražetina, Danijela Tipura, Maja Ćurčić, Ivan Miko, Antonija Mihelčić, Andrea Romić, Andrea Kukoč, Vanja Blagaj, Hrvoje Lasić, Emil Dolenc, Sonja Hleb, Hani Almahariq, Andrej Šribar, Jasminka Peršec, Ivica Lukšić

**Affiliations:** 1 Clinical Department of Anesthesiology, Reanimatology, and Intensive Care Medicine, University Hospital Dubrava, Zagreb, Croatia; 2 Zagreb University, School of Dental Medicine, Zagreb, Croatia; 3 Clinical Department of Maxillofacial and Oral Surgery, University Hospital Dubrava, Zagreb, Croatia; 4 Zagreb University School of Medicine, Zagreb, Croatia

## Abstract

**Aim:**

To describe epidemiological characteristics and baseline clinical features, laboratory findings at intensive care unit (ICU) admission, and survival rates of critically ill coronavirus disease 2019 (COVID-19) patients treated at a tertiary institution specialized for COVID-19 patients.

**Methods:**

This retrospective study recruited 692 patients (67.1% men). Baseline demographic data, major comorbidities, anthropometric measurements, clinical features, and laboratory findings at admission were compared between survivors and non-survivors.

**Results:**

The median age was 72 (64-78) years. The median body mass index was 29.1 kg/m^2^. The most relevant comorbidities were diabetes mellitus (32.6%), arterial hypertension (71.2%), congestive heart failure (19.1%), chronic kidney disease (12.6%), and hematological disorders (10.3%). The median number of comorbidities was 3 and median Charlson Comorbidity Index (CCI) was 5. A total of 61.8% patients received high-flow nasal oxygen therapy (HFNO) and 80.5% received mechanical ventilation (MV). Median duration of HFNO was 3, and that of MV was 7 days. ICU mortality rate was 72.7%. Survivors had significantly lower age, number of comorbidities, CCI, sequential organ failure assessment score, serum ferritin, C-reactive protein, D-dimer, and procalcitonin, interleukin-6, lactate, white blood cell, and neutrophil counts. They also had higher lymphocyte counts, PaO_2_/FiO_2_ ratio, and glomerular filtration rate at admission. Length of ICU stay was 9 days. The median survival was 11 days for mechanically ventilated patients, and 24 days for patients who were not mechanically ventilated.

**Conclusion:**

The parameters that differentiate survivors from non-survivors are in agreement with published data. Further multivariate analyses are warranted to identify individual mortality risk factors.

The first case of coronavirus disease 2019 (COVID-19) in Croatia was confirmed on February 25, 2020 (1). Very soon, on March 11, the World Health Organization (WHO) declared a COVID-19 pandemic (2). As of February 25, 2021, there were more than 240 000 confirmed cases and 5489 deaths in Croatia.

As a part of the national strategy against COVID-19 pandemic, the Ministry of Health and Civil Protection Headquarters decided that University Hospital Dubrava (UH) is to be repurposed into a Primary Respiratory Center for patients with confirmed COVID-19 infection. The intensive center of primary respiratory intensive center (PRIC-IC) is a subunit of UH Dubrava reserved for the treatment of patients with severe symptoms of COVID-19 who require mechanical ventilation, vasoactive hemodynamic support, continuous renal replacement therapy, and other aspects of intensive care (3). UH Dubrava became a COVID-19 tertiary center treating a third of all COVID-19 positive ICU patients in the country.

As the pandemic was surging through Europe, the number of critically ill COVID-19 patients in UH Dubrava continued to grow, and ICU capacities needed expansion. During winter months, six intensive care units in PRIC were operating at the same time: Three were run by intensivists from UH Dubrava and three by intensivists from other hospitals in Zagreb, including University Hospital Center Zagreb, University Hospital Center Sestre Milosrdnice, University Hospital Sveti Duh, University Hospital Merkur, and Children's Hospital Zagreb. The outcomes of critically ill patients treated in PRIC-IC therefore represent the work of intensivists from all hospitals in Zagreb.

Although scientific knowledge of COVID-19 increases daily, limited information is available regarding early identification of individuals who are at risk of developing severe symptoms. Previous studies reported certain demographic features and clinical characteristics of patients who were likely to develop severe symptoms of COVID-19 and thus require mechanical ventilation (4-7). Studies worldwide reported high mortality rates for patients requiring mechanical ventilation, ranging from 40% to 97% (4,8-10). Unfortunately, some of these reports were preliminary and included patients without a completed ICU stay. The aim of our cohort retrospective study is to describe the demographic characteristic, clinical features, laboratory values, and outcomes among critically ill COVID-19 patients treated in PRIC-IC, UH Dubrava.

## Patients and methods

We retrospectively reviewed the records of patients admitted to the combined intensive care unit (ICU) organized in a specialized PRIC-IC UH Dubrava between April 1, 2020 and February 1, 2021.

Data were collected from the hospital's information system (iBIS, IN2, Zagreb, Croatia). We recorded basic demographic characteristics (sex, age); laboratory parameters at ICU admission: white blood cell count (WBC, ×10^9^/L), neutrophil and lymphocyte percentage in white blood cells, the ratio of arterial oxygen partial pressure to fractional inspired oxygen (PaO_2_/FiO_2_) (mmHg), serum D-dimer (mg/L), serum lactate (mmol/L), serum ferritin (μg/L), serum procalcitonin (ng/mL), serum C-reactive protein (CRP, mg/L), serum interleukin-6 (pg/mL), and glomerular filtration rate (mL/min/1.73 m^2^); organizational aspects (whether the patients were admitted to the ICU from other departments of PRIC UH Dubrava or directly from ICUs in other hospitals in continental Croatia); body mass index (BMI, kg/m^2^); major comorbidities (arterial hypertension, diabetes mellitus, congestive heart failure defined as NYHA status>II, chronic kidney disease defined as glomerular filtration rate <60 mL/min/1.73 m^2^ calculated by using Chronic Kidney Disease Epidemiology Collaboration formula and chronic hematologic disorders); Charlson Comorbidity Index (CCI); sequential organ failure assessment (SOFA) score; duration of COVID-19 disease ICU admission; duration of ICU stay; mechanical ventilation; high-flow nasal oxygen therapy (HFNO); and ICU and in-hospital mortality rate. The study was approved by the Ethics Committee of UH Dubrava.

### Statistical analysis

Normality of distribution was assessed with the Shapiro-Wilk test. Continuous variables are displayed as either mean and standard deviation (SD) or median and interquartile range. Categorical variables are displayed as counts and percentages. The *t* test for independent samples or Mann-Whitney U test were used to test for differences between independent continuous variables, and χ^2^ or Fisher exact test for 2 × 2 tables were used to test for differences in categorical variables. Log-rank test was used to determine the difference in survival times between patient groups, and Kaplan-Meier plots were used to present the data. *P* values <0.05 were considered significant. The software used for statistical analysis and data visualization was jamovi, version 1.6.16 (11), and JASP, version 0.14.1 (12).

## Results

Between March 1, 2020 and February 1, 2021, 3736 patients were admitted to PRIC UH Dubrava because of COVID-19, and 692 (18.5%) of them were eventually admitted to PRIC-IC.

**Figure 1:**
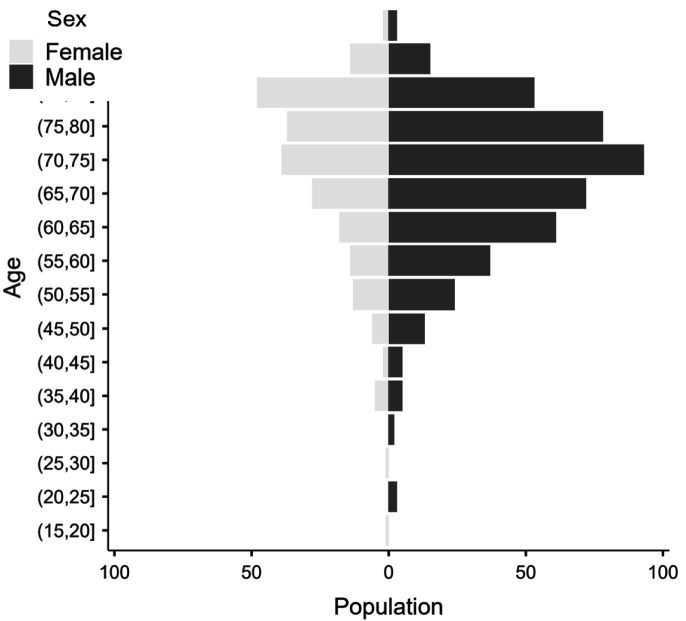
Age and sex distribution of critically ill coronavirus disease 2019 patients.

The median age was 72 (64-78) years. The study involved 464 men (67.1%) (Figure 1). The time from positive SARS-CoV-2 nasopharyngeal swab to the ICU admission was 5 (1-9) days. A total of 396 (57.2%) patients were admitted from the PRIC-respiratory center (ie, hospital wards) after worsening of clinical condition, while 297 (42.8%) patients were either transferred directly from ICUs in other hospitals in central Croatia or from UH Dubrava emergency department due to severe clinical presentation at hospital admission.

The median BMI was 29.1 (26.9-33.7) kg/m^2^. The most relevant comorbidities were diabetes mellitus (225 patients or 32.6%), arterial hypertension (492 or 71.2%), congestive heart failure (132 or 19.1%), chronic kidney disease (87 or 12.6%), and hematological (non-malignant) disorders (71 or 10.3%). The median number of comorbidities was 3 (2-4), and CCI was 5 (3-7). In terms of diagnoses affecting life expectancy, 75 (10.8%) patients had disseminated malignant disease and 56 (8.1%) were admitted from palliative care facilities.

In terms of clinical features and inflammation-related laboratory markers at ICU admission, SOFA score was 4 (2-5), WBC was 12.5 ± 7.4 × 10^9^/L with 10.8 ± 5.9 × 10^9^/L (86.1 ± 12.4%) neutrophils and 0.57 (0.37-0.86) × 10^9^/L (5.4% [3.1-8.9]) lymphocytes in differential blood count. CRP at admission was 137.9 ± 89.3 mg/L, procalcitonin was 0.55 (0.19-1.88) ng/mL, interleukin-6 was 67.9 (29.7-158.8) pg/mL, and serum ferritin was 1.0 (0.62-1.82) mg/L. Serum D-dimer at ICU admission was 3.17 (1.28-4.28) mg/L.

In terms of oxygenation and oxygen delivery and utilization parameters, PaO_2_/FiO_2_ ratio at ICU admission was 75 (56-125) mmHg, serum lactate levels were 1.6 (1.3-2.5) mmol/L, and glomerular filtration rate at admission was 78.8 (45.6-96) mL/min/1.73 m^2^.

In terms of organ support, 428 (61.8%) patients received HFNO, and 557 (80.5%) received mechanical ventilation. A total of 339 (79.2%) patients who received HFNO also received mechanical ventilation. Duration of HFNO was 3 (1-6) days and duration of mechanical ventilation was 7 (3-12) days. Thirty-six (5.2%) patients only received supplemental oxygen during ICU stay, and 6 patients (0.9%) received extracorporeal membrane oxygenation support. Forty-one patients (5.9%) received renal replacement therapy (RRT), 16 of them receiving intermittent hemodialysis (IHD), 18 continuous RRT (CRRT), 2 both IHD and CRRT, and 5 patients were dialyzed due to end-stage renal disease and need for chronic renal replacement therapy.

**Figure 2:**
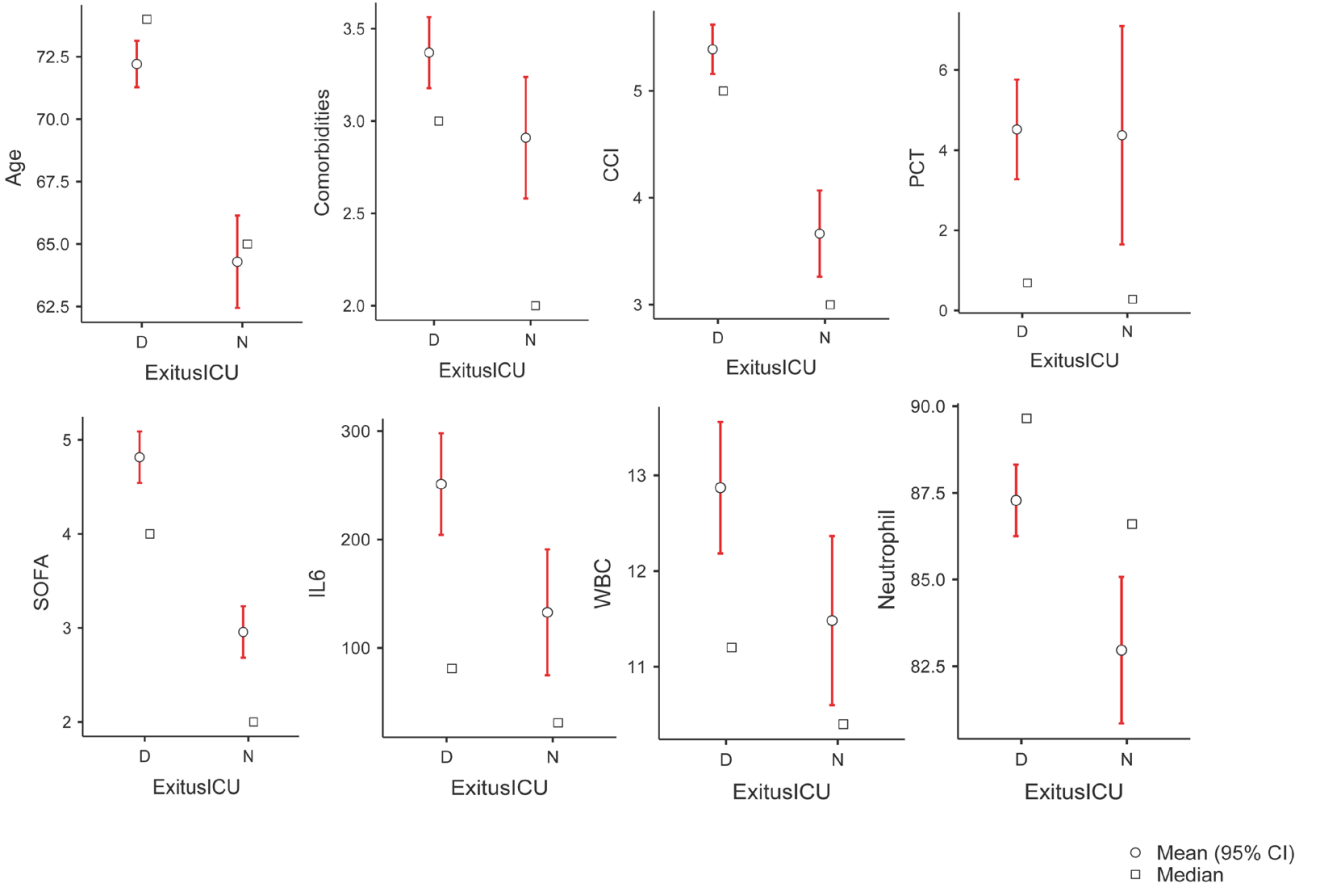
Age, number of comorbidities, Charlson Comorbidity Index (CCI), procalcitonin (PCT), sequential Organ Failure Assessment (SOFA) score, interleukin-6 (IL-6), white blood cell (WBC), and neutrophil percentage at intensive care unit admission in non-deceased (N) and deceased (D) critically ill coronavirus disease 2019 patients.

**Figure 3:**
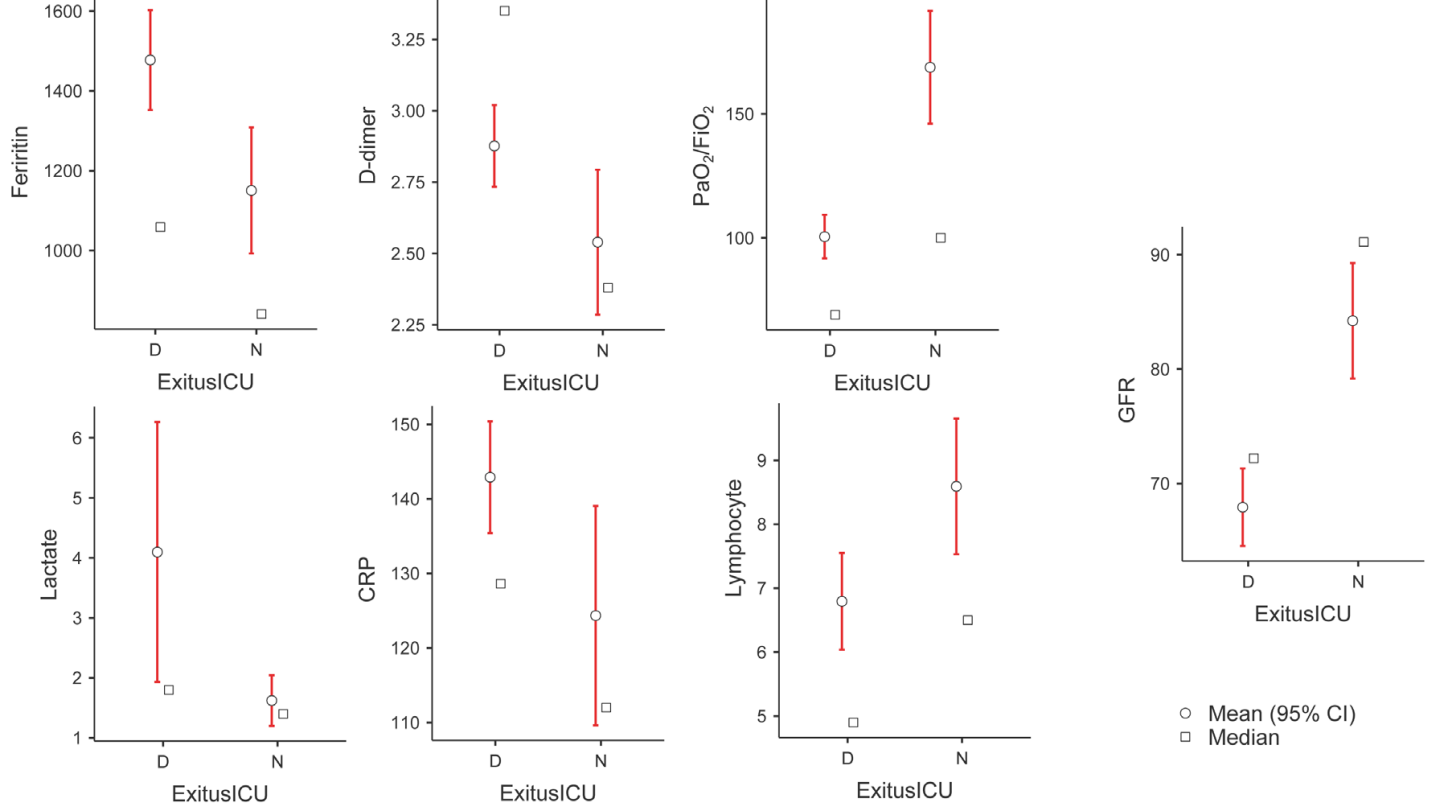
Serum ferritin, D-dimer, the ratio of arterial oxygen partial pressure to fractional inspired oxygen (PaO_2_/FiO_2_), lactate, C-reactive protein (CRP), lymphocyte percentage in white blood cell count, and glomerular filtration rate (GFR) at intensive care unit (ICU) admission non-deceased (N) and deceased (D) critically ill coronavirus disease 2019 patients.

Survivors had significantly lower age, number of comorbidities, CCI, SOFA score, serum ferritin, CRP, D-dimer, procalcitonin, interleukin-6, lactate, WBC and neutrophil counts; and higher lymphocyte counts, PaO_2_/FiO_2_ ratio, and GFR at admission (Figures 2 and 3). The groups did not significantly differ in BMI and days from a positive nasopharyngeal swab (Table 1).

**Table 1:** Baseline continuous variables in intensive care unit surviving and non-surviving critically ill coronavirus disease 2019 patients*

Variable	Survivors	Non-survivors	P*
Age (years)	65 (56-73)	74 (67-79)	<0.001
Body mass index (kg/m^2^)	31.2 ± 6.2	30.1 ± 5.6	0.411
Number of comorbidities	2 (1-4)	3 (2-4)	0.001
SARS-CoV-2 positive days	5 (1-8)	5 (2-9)	0.067
Charlson Comorbidity Index	3 (2-5)	5 (4-7)	<0.001
Sequential organ failure assessment	2 (2-4)	4 (2-6)	<0.001
Ratio of arterial oxygen partial pressure to fractional inspired oxygen (mmHg)	100 (70-224)	69 (55-103)	<0.001
Ferritin (mg/L)	1.15 ± 0.98	1.48 ± 1.06	0.003
D-dimer (mg/L)	2.54 ± 1.61	2.88 ± 3.35	0.013
C-reactive protein (mg/L)	112 (46-171)	129 (80-190)	0.001
Procalcitonin (ng/mL)	0.28 (0.10-0.82)	0.69 (0.24-2.5)	<0.001
Interleukin-6 (pg/mL)	31 (14-94)	81 (43-187)	<0.001
Lactate (mmol/L)	1.4 (1.1-1.8)	1.8 (1.3-3.4)	0.016
Glomerular filtration rate (ml/min/1.73 m^2^)	91 (61-106)	72 (41-92)	<0.001
White blood cells ( × 10^9^/L)	10.4 (7.7-14)	11.2 (8.2-16.4)	0.030
Neutrophil (%)	86.6 (80.3-90.9)	89.7 (85.8-92.9)	<0.001
Lymphocyte (%)	6.5 (4.1-10.7)	4.9 (2.9-8.3)	<0.001

*t-test or Mann-Whitney U test.

Out of 692 patients, 503 (72.7%) died during the ICU stay, and 25 after ICU discharge, with cumulative in-hospital mortality of 76.7%. The mortality rate was significantly higher in older age groups and in patients admitted from the ward after clinical deterioration (compared with those admitted directly from the emergency department or from ICUs in other hospitals). It was also higher in mechanically ventilated patients (with ICU mortality of 83.8%, opposed to 26.7% of deceased patients who did not receive mechanical ventilation, *P* < 0.001), as well as in those with diabetes mellitus, congestive heart failure, chronic kidney disease, and bacterial superinfections (81.2% ICU mortality rate in patients who developed bacterial superinfections, compared with 62.1% mortality rate of those who did not develop bacterial superinfections, *P* < 0.001). There were no significant differences in ICU mortality rates between female and male patients, and between patients with hematologic comorbidities, arterial hypertension, myocardial or cerebrovascular infarction and patients who did not have these conditions (Table 2). RRT, HFNO, and ECMO were not linked to significant differences in survival rates.

**Table 2:** Categorical variables in intensive care unit surviving and non-surviving critically ill coronavirus disease 2019 patients*

		No. (%) of		
Variable		survivors	non-survivors	P
Sex	M	127 (27.4)	337 (72.6)	0.961
	F	62 (27.2)	166 (33.8)	
Arterial hypertension	yes	124 (25.2)	368 (74.8)	0.073
	no	64 (32.2)	135 (67.8)	
Diabetes mellitus	yes	46 (20.4)	179 (79.6)	0.006
	no	142 (30.5)	323 (69.5)	
Chronic kidney disease	yes	14 (16.1)	73 (83.9)	0.014
	no	174 (28.8)	430 (71.2)	
Congestive heart failure	yes	24 (18.2)	108 (81.8)	0.009
	no	164 (29.3)	395 (70.7)	
Hematologic disease	yes	13 (18.3)	58 (81.7)	0.091
	no	175 (28.2)	445 (71.8)	
Ward admission	yes	71 (17.9)	325 (82.1)	<0.001
	no	118 (39.9)	178 (60.1)	
Bacterial superinfection	yes	72 (18.8)	311 (81.2)	<0.001
	no	117 (37.9)	192 (62.1)	
Myocardial infarction/cerebrovascular infarction at admission	yes	13 (25)	39 (75)	0.871
	no	175 (27.4)	463 (92.2)	
Age (whole cohort)	<45	12 (57.1)	9 (42.9)	<0.001
	45 - 65	78 (45.6)	93 (54.4)	
	65 - 75	56 (24.8)	170 (75.2)	
	>75	43 (15.7)	231 (84.3)	
Age (mechanically ventilated)	<45	7 (46.7)	8 (53.3)	<0.001
	45-65	36 (29.5)	86 (70.5)	
	65-75	23 (12.4)	163 (87.6)	
	>75	22 (9.6)	207 (90.4)	

*χ^2^ or Fisher exact test for 2 × 2 tables.

**Figure 4:**
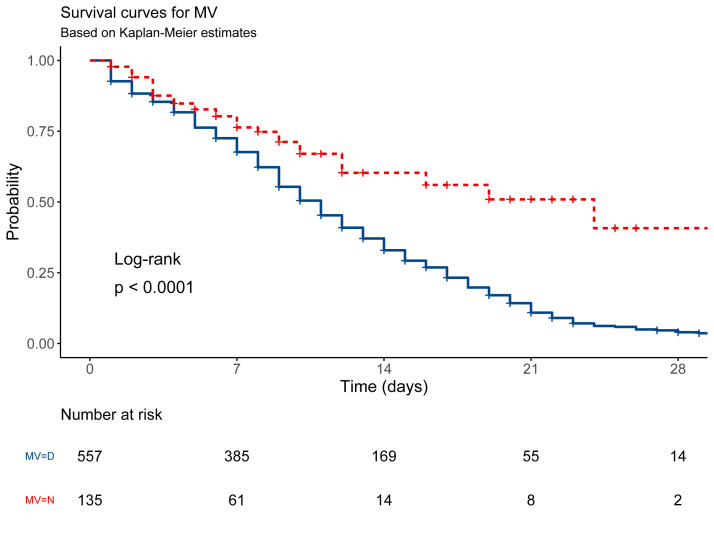
Kaplan-Meier survival curve depicting 28-day survival probability depending on the need for mechanical ventilation. Dotted line represents patients who did not receive mechanical ventilation.

**Figure 5:**
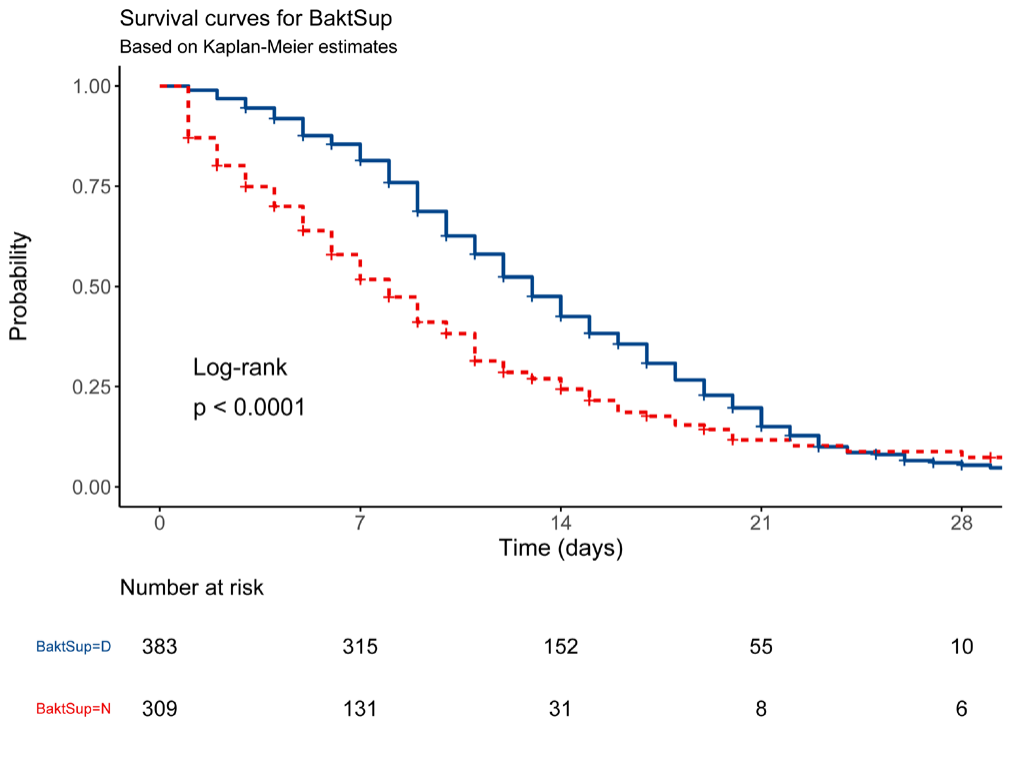
Kaplan-Meier survival curve depicting 28-day survival probability depending on bacterial superinfection rate. Dotted line represents patients who did not develop bacterial superinfection.

Length of ICU stay was 9 (4-14) days. The median survival was 11 days for mechanically ventilated patients, and 24 days for patients who were not mechanically ventilated (Figure 4). The median survival was 13 days for patients with bacterial superinfections and 8 days for patients without bacterial superinfections (Figure 5).

## Discussion

In this study, old age, diabetes mellitus, chronic kidney disease, and congestive heart failure were identified as significant risk factors for ICU and in-hospital mortality. The majority of analyzed patients were admitted to the ICU in an advanced state of disease with severe clinical presentation (bilateral pneumonia complicated with severe ARDS and dysregulated inflammatory response) and deranged laboratory findings (elevated serum D-dimer levels and lower lymphocyte count).

Wu et al (4) reported similar risk factors and laboratory values among the first 201 patients in Wuhan, but their cohort included only 26.4% of ICU patients. They also identified older age, neutrophilia, lymphopenia, higher interleukin-6, and D-dimer levels as significant risk factors in patients with acute respiratory distress syndrome (4). Auld et al (9) published similar laboratory values (SOFA score, Horowitz quotient, CRP, and D-dimer levels) but in our study the prevalences of congestive heart failure and diabetes mellitus were significantly higher in non-survivors.

Most of the previous studies recruited patients till June 2020, during the so-called “first pandemic wave,” when COVID-19 patients had significantly lower-case fatality rate, at least in Croatia. Most risk factors that we identified are consistent with those identified in other retrospective studies (5-7).

In several studies, older age was identified as a risk factor both in univariate and multivariate analyses (13-20). Our patients, with a mean age of 72 years, are the oldest among all reported ICU populations known to authors at the time of writing. In all ICU reports, there was a predominance of male participants, and Rodriguez et al (13) and Xie et al (21) observed higher survival rates in women.

The majority of our patients (71.2%) had hypertension before ICU admission, but no significant difference was observed between survivors and non survivors, unlike in other studies (13,15,19,21). The prevalence of hypertension in ICU patients varied from 38% (15) to 78% (22). A recent meta-analysis (23) showed that in SARS-CoV-2-positive patients, hypertension was associated with almost 2.5-fold higher mortality risk.

In our study, the prevalence of diabetes was significantly higher among non-survivors, which is consistent with several similar studies (13,15,19). A meta-analysis including 16 003 patients found that diabetes mellitus was significantly associated with COVID-19 mortality, with a pooled odds ratio of 1.90 (24). Several pathophysiological mechanisms that occur in diabetes mellitus can facilitate deterioration of COVID-19, such as chronic inflammatory state, inhibited lymphocyte proliferative response, imbalance between coagulation and fibrinolysis, endothelial dysfunction, and enhanced platelet aggregation and activation (25).

In our study, chronic kidney disease was significantly more prevalent in non-survivors. This was also reported by Rodriguez et al (13). An association between chronic kidney disease and severe COVID-19 was observed in a meta-analysis by Henry and Lippi (26), with a pooled odds ratio of 3.03. A single-center study from Madrid reported CKD before hospital admission as a risk factor for in-hospital mortality (27). Two large multicentric studies identified higher renal components of SOFA score at ICU admission as independent risk factors for mortality (19,28).

In our study, non- survivors more often had chronic congestive heart failure. Two studies from New York City found chronic heart failure to be a risk factor for increased ICU mortality (17,18). Interestingly, in a retrospective study from Madrid, patients with underlying chronic heart failure were less often admitted to the ICU, but their mortality rates were significantly higher than those of patients without this condition (29).

Our study also reported that non-survivors had significantly lower lymphocyte count, which was also observed in several previous studies (19-21,30). A meta-analysis including 1289 patients reported a significant correlation between lymphopenia and COVID-19 severity (31). The trend in relative lymphocyte count was evaluated as a good prognostic tool for the disease severity (32). The same article proposed several pathophysiological mechanisms by which SARS-CoV-2 virus could cause lymphopenia: direct cytotoxic effect, direct effect on lymphoproliferative organs, and cytokine storm that could be responsible for lymphocyte dysfunction and apoptosis (32).

Survivors also had lower CRP, procalcitonin, ferritin, and interleukin-6 levels. CRP and interleukin-6 can be used as predictors of disease severity (33) and the need for mechanical ventilation (34). Procalcitonin is a helpful tool to identify a superimposed bacterial infection in COVID-19 patients (33). A meta-analysis of 18 studies revealed a role of ferritin in indicating a severe disease in 4992 COVID-19 patients and a mortality risk in 2621 patients (35).

Although crude survival rates were lower in both ventilated patients and those who developed bacterial superinfections, patients with bacterial superinfections had longer ICU stay. This finding could be explained by the response to corticosteroid therapy, which is a part of the therapeutic regimen according to hospital’s COVID-19 pharmacological guidelines. Corticosteroid therapy delayed early death from SARS-CoV-2-induced cytokine storm during the early phase of ICU stay, but put patients at risk for sepsis-induced organ damage and death later in the ICU stay.

Previous studies reported increased D-dimer levels in ICU non-survivors (13,18,19). A study including 343 patients from an emergency department found that D-dimer levels above 2.0 μg/mL predicted in-hospital mortality (36). In comparison with previously published data (13,18,19), our patients had the second highest reported D-dimer levels (3.17 [1.28-4.28] mg/L). Furthermore, our laboratory is able to measure only the levels as high as 4.45 mg/L or 4.23 mg/L depending on the assays and reagents used (higher values are reported with a “greater than” sign). Somewhat less than a third of our patients had D-dimer above this level, so that the real median D-dimer levels were very likely markedly higher than the reported value.

Finally, the PaO_2_/FIO_2_ ratio at admission (median of 75 mm Hg) was the second worst reported value, after that in 52 ICU patients in Wuhan (16). According to the Berlin definition, this value could be classified as severe ARDS (37), with an expected decrease in survival rates (38).

The majority of our results were obtained in the second pandemic wave, when the Croatian health care system was faced with an enormous burden. This was especially the case in comparison with the first, smaller wave, which lasted till May 2020 and only included the first 65 patients from this cohort (39). Furthermore, in December 2020, two earthquakes hit the town of Petrinja in Sisak-Moslavina County. Most of the ICU patients from damaged hospitals in the surrounding area were urgently transferred to our clinic. This created not just a fast influx of patients but also increased the risk of new and different multi-resistant bacterial strains.

Our study has several limitations. First, it was performed in a very specific single-center setting of the national COVID-19 center treating a third of all SARS-CoV-2 positive ICU patients in the country. However, this study is in practice a multi-center study since PRIC-UH Dubrava had 6 ICUs with critical care physicians and nurses from all hospitals in Zagreb, and with different workflows, protocols, and ICU charts. Second, this center had to deal with indirect effects of another natural disaster, which could easily have affected the reported complication and survival rates.

In conclusion, since the start of COVID-19 pandemic in Croatia, PRIC-UH Dubrava has become the largest COVID-19 hospital in Croatia with the greatest patient turnaround. Due to the heterogeneity of patients included in this cohort and due to differences in clinical presentations in the subsequent pandemic waves, further multivariate analyses are needed to identify risk factors affecting the survival rates of critically ill COVID-19 patients.
